# What We Know and What Remains to Be Explored about LGBTQ Parent Families in Israel: A Sociocultural Perspective

**DOI:** 10.3390/ijerph19074355

**Published:** 2022-04-05

**Authors:** Geva Shenkman, Dorit Segal-Engelchin, Orit Taubman–Ben-Ari

**Affiliations:** 1 Baruch Ivcher School of Psychology, Reichman University (IDC Herzliya), Herzliya 4610101, Israel; 2 The Spitzer Department of Social Work, Ben-Gurion University of the Negev, Beer-Sheva 84105, Israel; dorsegal@bgu.ac.il; 3 School of Social Work, Bar-Ilan University, Ramat-Gan 5290002, Israel; taubman@biu.ac.il

**Keywords:** LGBTQ parent families, same-sex parents, gay fathers, lesbian mothers, Israel

## Abstract

This paper reviews research on gay and lesbian parent families in Israel through cultural lenses while recognizing the diversity of these families. The major aims of the review are: (1) to provide an overview of the situation of LGBTQ parent families in Israel, as well as of the sociocultural background of the Israeli context and its effects on sexual minorities and LGBTQ parent families; and (2) to identify the limitations and lacunas in the existing research and shed light on what remains to be explored. We searched numerous databases for relevant studies, adopting a narrative approach to summarize the main findings while taking into account the literature on the socio-cultural context in Israel and its impact on sexual minorities and LGBTQ parent families. The search yielded empirical results only for gay and lesbian parent families, with studies emphasizing the challenges they face and the factors related to their well-being and that of LGB individuals aspiring to become parents. In addition, it revealed that research on children’s psychosocial adjustment as a function of parental sexual orientation is quite scarce in Israel. Moreover, it indicated the absence of investigations of bisexual, transgender, or queer parents. We conclude that the sociocultural context of Israel, including its pronatalist and familistic orientation, may play an important role in shaping the experiences of LGBTQ parent families, and should be taken into consideration when studying LGBTQ parents.

## 1. Introduction

Today, more than ever before, the number of families headed by lesbian and gay parents is growing worldwide [1,2,3,4,5]. At the same time, the legal climate surrounding the civil rights of sexual and gender minorities, as well as attitudes towards diverse family forms, are becoming more accepting [6]. Nevertheless, there is still considerable heterogeneity between countries in regard to laws concerning LGBTQ parent families, ranging from full legal recognition to criminalization [7]. This heterogeneity may impact child and parent well-being [7]. Consequently, researchers have underscored the need to focus more attention on studying LGBTQ parent families from a sociocultural perspective, e.g., [7,8].

In recent decades, a large body of research has been conducted on diverse LGBTQ parent family forms, including lesbian and gay stepfamilies formed post heterosexual relationship dissolution [9], lesbian-parent families through donor insemination [2], LGBTQ parent families created by adoption [10], shared parenting arrangements between gay and lesbian individuals [11], and gay men becoming fathers through surrogacy [12]. The topics investigated in the existing research range from child adjustment in LGBTQ parent families to the well-being of the parents, with results showing that parents’ sexual orientation does not have a detrimental effect on the adjustment of either sexual minority parents or their children, e.g., [13,14,15]. Research on child adjustment among LGBTQ parent families showed that children in these families did not differ from children of heterosexual parents in performance at school, academic achievements, physical health, emotional and behavioral difficulties, peer relations, or atypical gender role behavior, e.g., [15]. They also did not differ in internalizing or externalizing problems from children of heterosexual parents, e.g., [12]. Furthermore, increased parenting attention for children has been reported among gay and lesbian families, which may promote their educational achievements and self-esteem later in life, e.g., [2,6].

The current paper seeks to further our understanding of the sociocultural impact on LGBTQ parent families by considering those living in Israel. The role of sociocultural impact is viewed through the cross-cultural theoretical perspective, which suggests taking into account the specific cultural context, alongside the symbolic systems of beliefs and norms surrounding sexuality, as these may help in better understanding both the unique strengths and the specific challenges faced by LGBTQ parent families [16]. While reviews of the literature on LGBTQ parent families have been conducted in other countries, e.g., [17], no previous review has considered the relevant literature in Israel or addressed pertinent sociocultural parameters characterizing Israeli society such as pronatalism and familism when reviewing the accumulated literature. We believe this is a very apt time to review the research on LGBTQ parent families in Israel because of the recent Supreme Court ruling in July 2021 requiring a change in all definitions in the current law that prevent the access of gay men to surrogacy services in Israel within six months [18]. This ruling, which reflects a more positive attitude toward gay parenting, may encourage many more gay men in Israel to consider the possibility of becoming parents and may enable them to pursue fatherhood in a more open and less discriminating environment. Thus, this is a particularly appropriate moment to reflect on the picture that emerges from research conducted before the profound change in legislation.

Although research on LGBTQ parent families in Israel has increased in recent decades, no review of existing studies has been conducted. Accordingly, our review has two objectives. First, to provide an overview of the situation of LGBTQ parent families in Israel and the sociocultural background of the Israeli context and its effects on sexual minorities and LGBTQ parent families. Secondly, to identify the limitations and lacunas in the research, and shed light on what remains to be explored. Following these objectives, in our review, we first explore the social, contextual, religious, and legal factors that may impact LGBTQ parent families and LGBTQ individuals aspiring to become parents in Israel. We then review the empirical literature addressing gay and lesbian parent families. Finally, we offer suggestions for future research, with an emphasis on the contribution of sociocultural factors. Notably, in order to present a more inclusive perspective, we use the term “LGBTQ parent families” in this paper, while specifying the particular population sampled in each study.

## 2. The Sociocultural Context of Israel and Its Relation to LGBTQ Parent Families

The sociocultural context of Israel offers a unique setting for studying LGBTQ parent families as it strongly promotes the value of childbearing while simultaneously placing numerous legal obstacles in the way of gay men who wish to become parents. On the one hand, Israel is considered a familistic and pronatalist society cherishing child-rearing [19,20,21], manifested in the highest fertility rates of all Organisation for Economic Co-operation and Development (OECD) countries [22]. Childbearing and parenting are highly esteemed, so that being a parent is regarded as the main pathway to social acceptance [23,24]. The Biblical commandment to ”be fruitful and multiply,” the openness of Jewish religious authorities to assisted reproductive technologies (ART), and the traumas of the Holocaust have all been proposed as contributors to the culture of familism and the high birth rate [25]. Moreover, child-oriented benefits, such as birth allowances and tax deductions based on the number of children in the family, are long-standing features of government policy [23]. In addition, all women in Israel, whatever their marital or family status, are entitled to extensive Health Maintenance Organization (HMO) coverage for practically unlimited in vitro fertilization (IVF) cycles up to the age of 45 [21].

On the other hand, Israeli legislation places restrictions on LGBTQ individuals, and in particular gay men who wish to become parents. While several laws have been passed regarding the rights of LGBTQ couples, same-sex marriage in Israel is still impossible [26,27], and, until recently, surrogacy services in Israel were illegal for same-sex couples, although the law allowed for surrogacy for heterosexual couples or single women who were unable to conceive [28,29]. Thus, gay men who desired to become parents via surrogacy were forced to turn to highly expensive surrogacy services overseas, mainly in South East Asia and North America [29]. Attempts in recent years to expand access to surrogacy to the LGBTQ community have faced intense opposition from ultra-Orthodox Jewish political parties. Moreover, a 2018 law that extended eligibility to unmarried women started nationwide protests from the gay community and its supporters for excluding gay men. Nevertheless, in the same year, the Israeli Knesset (parliament) voted to reject a bill extending surrogacy laws to same-sex couples. In 2020, Israel’s Supreme Court ruled that the surrogacy laws discriminated against single men and gay couples and ordered the Knesset to enact a new law within a year. Arguing that it was unable to do so due to political contention, the Knesset asked the Supreme Court to take action itself. Consequently, in July 2021, the Supreme Court ruled that all definitions in the current law that prevent access to surrogacy services for gay men be changed within six months [18].

When addressing the challenges posed by the Israeli sociocultural and legal context for LGBTQ individuals, Orthodox Jewish law must also be taken into consideration. Strongly influenced by Biblical law, it disapproves of homosexuality, continuing to condemn and stigmatize LGBTQ individuals, thereby creating an atmosphere that is often perceived as hostile to sexual minorities [30,31]. Furthermore, it was only in 1988 that the Knesset repealed the British Mandate regulation that made homosexuality a criminal offense [32]. In addition, Israel is largely a patriarchal society that still adheres to masculine stereotypes, nourished by several years of army service that are mandatory for almost all men and women from the age of 18 [33]. In sum, it is challenging to become an LGBTQ parent in this societal context of traditional masculine roles, reliance of Jewish religion on the Biblical law that denigrates homosexuality, and legislation that does not allow same-sex marriage in the country, and, until recently, restricted access to local surrogacy services [28,34,35,36]. Adoption opportunities are also extremely limited, as newborns are rarely available for adoption in Israel in general, and there are few international adoption opportunities for LGBTQ people as well [26,37]. Alongside these multiple difficulties, powerful socialization processes of familist and pronatalist values may create social pressure for LGBTQ individuals, who are likely to internalize these messages. As a result, they may develop a strong desire to become a parent, while at the same time being highly aware that they will have to overcome daunting challenges and obstacles in order to fulfill this desire [38].

## 3. Methodology

This paper reviews research on LGBTQ parent families in Israel through cultural lenses. The search strategy that was used in our review aimed to locate the relevant studies, therefore we conducted searches in numerous databases through October 2021, including PsycNET, Proquest, Ebsco, Sociological Abstracts, and Google Scholar, using several combinations of keywords: “LGBTQ parents,” “same-sex,” “same-gender,” “gay,” “lesbian,” “queer,” and “bisexual” together with “families,” “parents,” “fathers,” “mothers,” “parenting,” “parenthood,” “Israel,” and “aspirations,” both in English and Hebrew. We also searched reference lists within published papers. Empirical studies (i.e., meta-analysis, qualitative, quantitative, and mixed methods studies) published in peer-review articles and books were eligible for inclusion in our review. Studies presented in unpublished doctoral dissertations and master theses were excluded from our review. As we aimed to detect all relevant papers, we did not limit the year of publication.

Although we followed the main steps for a systematic review, what we present here is not merely a review of the literature on LGBTQ parent families in Israel per se, as we also included studies on the sociocultural and legal context and its relation to sexual minorities and parenting in order to provide for a deeper understanding of the experience of Israeli LGBTQ parent families and the challenges they face. Furthermore, since both quantitative and qualitative studies were reviewed to produce a comprehensive picture, the disparity in the nature of the results may not be easily understood within the confines of a strictly systematic review. We, therefore, adopted a more narrative approach to summarize the main findings, as this appeared to be the best way to present the results from disparate studies while also addressing issues pertaining to the sociocultural and legal context.

## 4. Results

### 4.1. Parenthood Aspirations among Israeli LGBTQ Individuals

Nowadays, more LGBTQ individuals are becoming parents than ever before due to changes in legislation and advances in fertility technologies [39,40]. Consequently, research on parenthood aspirations in this population has been growing worldwide, e.g., [41,42,43,44,45], including in Israel, e.g., [38,46]. Previous studies have suggested that there are three main common factors that define whether an individual will actually become a parent in the future: parenthood desire (i.e., expressed wishes); parenthood intention (i.e., explicit reflecting/planning to become a parent); and estimated likelihood of parenthood (i.e., assessing the probabilities of becoming a parent, which also takes into account the sociocultural conditions) [38,47,48]. We found six Israeli studies addressing these issues. The first examined the frequencies of the desires and likelihood estimations of Israeli gay men regarding fatherhood, using a sample of 183 childless gay men aged 19–50 [38]. Results revealed a significant gap between strong fatherhood desires and low likelihood estimations. The authors suggest that this gap may be attributed to the Israeli context, with the results indicating tension between the internalization of dominant familist and pronatalist values on the one hand, which may explain the strong parenthood desires, and awareness of the multiple hurdles to gay parenthood in Israel on the other, which may account for the low likelihood estimations [21,38].

Other studies explored the difference in parenthood aspirations between LGB and heterosexual childless individuals. The first compared 395 Israeli lesbian women and gay men with 488 heterosexual participants and found that lesbian women and gay men reported less desire to be parents than their heterosexual counterparts [49]. The second focused on estimated likelihood of parenthood, comparing 202 lesbian women and gay men with 247 heterosexual individuals [46]. Results showed that lesbian women and gay men reported a lower estimated likelihood of parenthood than their heterosexual counterparts and that gay men reported the lowest estimations of future parenting. In addition, a lower estimated likelihood of parenthood was related to more depressive symptoms and lower happiness and satisfaction with life. The differences between the two groups echo similar findings in other countries, which are usually explained as resulting from stronger social pressure on heterosexual couples than LGBTQ individuals to have children and obey traditional gender roles [50], alongside LGB people’s awareness of the legal and social hurdles that could hinder their parenthood aspirations, e.g., [51,52]. Lower parenthood aspirations among LGB individuals could also stem from discrimination and prejudice on the part of reproductive health professionals [53], internalization of homonegativity [47], institutional discrimination such as state bans on same-sex marriage and adoption, and continuing minority social stress [8,54]. In a further attempt to explain the difference in parenthood aspirations between LGB and heterosexual individuals, a third study compared 174 Israeli LGB with 438 heterosexual participants, exploring the possible mediation of anticipation of stigma upon parenthood in the association between being a member of a sexual minority and lower parenting desires, intentions, and likelihood estimations [48]. Results revealed that LGB individuals reported higher anticipation of stigma upon parenthood and lower parenthood aspirations than their heterosexual counterparts. Moreover, the anticipation of stigma upon parenthood fully mediated the association between belonging to a sexual minority and lower parenthood desires and intentions, and partially mediated the association between belonging to a sexual minority and lower estimated likelihood of parenthood. These findings were understood in light of possible mindfulness of prejudice and discriminatory legislation that prohibited or impeded sexual minorities from pursuing parenthood.

Another study focusing on LGB parenthood aspirations investigated the mediation of lower parenthood aspirations in the association between being an LGB individual and negative mental health outcomes [55]. The sample consisted of 150 LGB and 377 heterosexual participants who completed questionnaires assessing parenthood aspirations, life satisfaction, and depression. It was found that lower parenthood aspirations fully mediated the association between being an LGB individual and lower satisfaction in life, and partially mediated the association between being an LGB individual and depressive symptoms. The findings shed light on the role of parenthood aspirations in mental health differences between LGB and heterosexual individuals, indicating a link between lower parenthood aspirations and adverse mental health in a familistic and pronatalist setting such as Israel.

In an examination of the contribution of different sociocultural contexts to parenthood aspirations, one study compared Israeli, Portuguese, and British childless LGB and heterosexual adults [56]. A sample of 168 participants from each country were individually matched on sociodemographic variables and compared on parenthood aspiration indicators and attitudes toward staying childfree. Results indicated that participants from Israel and Portugal reported higher levels of parenthood desire, intent, and concern about childlessness than participants from the United Kingdom. Parallel patterns also emerged among the countries for LGB and heterosexual participants separately. In addition, LGB participants, in general, reported lower levels of parenthood desire, intentions, and concern about childlessness than did heterosexual participants. The findings were explained in terms of the different sociocultural contexts of the countries, that is, the individualistic values characterizing the UK versus the familistic values in Israel and Portugal, along with the strong pronatalist stance in Israel and the economic context in Portugal. This study suggests the need for further cross-cultural comparisons in order to develop a more comprehensive view of parenthood aspirations, sexual orientation, and diverse sociocultural contexts.

### 4.2. Research on LGBTQ Parent Families in Israel

Being a parent (of any kind) is a path towards acceptance by a society that sanctifies family values and continuity [57]. In the case of the LGBTQ population, parenthood can be considered a victory over the prevalent message that LGBTQ individuals are not meant to be parents [58]. It has thus been suggested that successfully overcoming the social, legal, and financial obstacles on the expedition to parenthood in Israel might result in better subjective well-being (SWB). Erez and Shenkman [59], for example, compared 90 Israeli gay fathers with 90 individually matched heterosexual participants and found that after controlling for socio-demographic differences between the groups, gay fathers reported significantly higher levels of SWB than heterosexual fathers. Similarly, Shenkman and Shmotkin [60] compared 45 gay fathers with 45 individually matched gay men who were not fathers on indicators of SWB, depression, and life meaning. Results indicated that gay fathers reported higher levels of SWB and life meaning, as well as lower levels of depression, than gay men who were not fathers. This is a deviation from the pattern found in heterosexual samples, in which being a parent was related to decreased levels of SWB and increased levels of life meaning, e.g., [61].

Additional research also suggests positive psychological outcomes among Israeli gay fathers as compared to heterosexual fathers. In a study by Shenkman and Shmotkin [62], 82 Israeli gay fathers who became fathers, mainly through surrogacy, were individually matched with 82 heterosexual fathers, and were compared on life meaning and self-perceived parental role, that is, parents’ subjective assessments of their competence, investment in parenthood, and self-efficacy. Results showed that only among gay fathers was higher self-perceived parental role linked to higher life meaning. Furthermore, only among gay fathers was self-perceived parental role linked with less adverse mental health indicators (depression, negative emotions, and neuroticism) [19]. In another study, which examined middle-aged and older Israeli gay men, 76 who had become fathers in a previous heterosexual relationship were compared with 110 who were not fathers, and 114 heterosexual fathers [63]. Results showed that self-reported personal growth was higher among gay fathers than among heterosexual fathers after controlling for differences in socio-demographics between the groups. It was suggested that gay fathers, who were previously in a heterosexual relationship and currently identify themselves as exclusively gay, most likely had to overcome numerous hurdles as part of the multifaceted process of coming out to themselves, their spouse, and their children. Successfully coping with these hurdles might have resulted in the creation of new meaning in life, which might explicate the higher levels of personal growth found among older gay fathers as compared to heterosexual fathers. In addition, both personal growth and purpose in life were higher among gay fathers than among gay men who were not fathers. Taken together, these findings suggest that succeeding in becoming a gay father in Israel, which, as we have seen, is a familistic society that promotes childrearing but also places difficulties in the path of gay men who wish to become fathers, may lead to a stronger sense of meaning in life and SWB. Another comparative study employed a sample of 76 gay men who had become fathers through a heterosexual relationship, 63 gay men who had become fathers through surrogacy, and 78 heterosexual fathers, examining psychological well-being as indicated by life satisfaction, parenthood satisfaction, depressive symptoms, and the Big Five personality dimensions [64]. Results showed that after controlling for socio-demographic differences between the groups, gay fathers through surrogacy reported greater satisfaction with parenthood, greater satisfaction with their lives, and higher levels of extraversion than heterosexual fathers. No significant differences emerged between the three groups on depressive symptoms, neuroticism, conscientiousness, agreeableness, and openness to experience. This was a pioneering study in that it included both a comparison with heterosexual fathers (a between-difference approach) and a comparison between two pathways and experiences of gay fatherhood (a within-difference approach). The findings specifically depicted gay fathers through surrogacy as more extraverted and more satisfied with both their parenthood and their life in general. This was interpreted as possible resiliency manifestations, meaning that in a familistic and pronatalist society such as Israel, success in becoming a gay father after contending with the unique difficulties relating to overseas surrogacy, might result in elevated psychological well-being. Higher extraversion may imply the need to adopt active coping strategies when perusing surrogacy abroad [64].

In addition to quantitative studies examining the positive psychological outcomes of SWB and growth among gay fathers, qualitative studies have explored both the motivations for choosing different routes to parenthood and the reproductive experience of gay fathers. Erera and Segal-Engelchin [20] conducted in-depth interviews with nine Israeli gay fathers who were co-parenting with heterosexual women while maintaining separate households. This option for parenthood is fairly common in Israel and reflects traditional cultural values that privilege different-sex parent families [65,66]. The interviews revealed the main motivations for creating a hetero-gay family, including the belief in the essential need for a mother, the belief in biological connection to the child, and the belief that the child’s best interests dictate having two different-sex parents. Another qualitative study of 16 Israeli gay men expecting a child through surrogacy overseas, mostly in India, explored the emotional experience of pregnancy [67]. It was found that the men often felt frustration and concern due to their distance from the pregnant woman and, specifically, their inability to experience the physical presence of the fetus, which hindered the development of their parental identity during the pregnancy. Similarly, Lustenberger [68] described the experience of a group of gay men who contracted with surrogate mothers in Mumbai and traveled to the Indian city for the birth. The main theme emerging from the interviews was the bureaucratic hardships involved in transnational surrogacy. The men reported that the bureaucratic procedures seldom went smoothly, leading to many moments of insecurity and standstill. Lustenberger stresses that the ability to recruit the necessary capital for lawyers and medical agents to resolve the bureaucratic problems indicates the privileged status of her sample and that without access to substantial financial resources, the capacity of gay men to become parents is dramatically curtailed. Furthermore, she argues that the narratives of Israeli gay men who pursue fatherhood in India provide important insight into the dynamics between vulnerability and privilege.

Another qualitative study interviewed 39 Israeli gay men who became fathers through surrogacy [24]. The authors suggest that gay parenthood undermines existing concepts of parenthood, especially the essentialist notion of motherhood as a social construct resulting from an innate order. Essentialist notions of motherhood reflect heteronormative ideologies and the demand for the presence of parents of both genders. In addition, the authors contend that the parenting experience of gay men who become fathers through surrogacy is shaped by contradictions. Such contradictions include the tension between biogenetic and social concepts of parenthood and kinship, as one of the fathers is usually not genetically related to the child. Another contradiction refers to the dialectic between continuity and change, as gay fathers construct ”family,” which is the main indicator of social inclusion, in a way that obeys central pronatalist ideology, while simultaneously defying the traditional definition of family as consisting of a father and mother. These contradictions may impact gay parents’ identities and the construction of autonomy concepts in their lives, as they struggle to hold together the duality between social acceptance and subversion [57,69]. In another qualitative study, the experience of 14 Jewish-Israeli gay fathers who became parents through surrogacy overseas was explored as a function of spatial notions [70]. The authors maintain that gay fathers living in the periphery of the country challenge the dominant monolithic perception of this area as a locus of homophobia, thus expanding the boundaries of the “center” to include “marginalized” cultural entities, roles, and identities such as gay fatherhood. Taken together, the findings from these studies mirror the duality of gay fathers’ parenting experience, which is molded by the heteronormative hegemonic discourse on parenthood, center, and periphery, while at the same time contradicting its gendered and binary attitudes.

In a further qualitative study, 60 in-depth interviews were conducted with Israeli gay men who became fathers through a variety of means (adoption, surrogacy, or co-parenting as singles, couples, or multi-parent families) [71]. The study explored the experience of parenthood through social scripts, i.e., the socially expected order of actions that are derived from self-explanatory norms in a society or subculture at a given time, employing a sample representing a wide range of ages (from the late twenties to age sixty). Results showed that during the 1970s and 1980s, counter-hegemonic scripts emerged that rejected the heteronormative ideal of natality. Then, in the 1990s, a different script emerged that separated parenthood from couple-based relationships and allowed for separate co-parenthood relationships. Since 2005, with the introduction of surrogacy as a medical option that can be legally pursued overseas, the previous discourses have waned and been substituted by a new couple-based fatherhood script. The authors suggest that in Israel, a pronatalist society that embraces medically assisted reproduction, surrogacy offers a new form of normative gay parenthood.

In view of this understanding, 65 qualitative in-depth interviews were conducted with self-identified LGB individuals in a stable relationship. All the couples, save for four, were parents [72]. The study drew on findings from extended ethnographic research conducted between 2010 and 2012 on the formation of same-sex parenthood in Jewish-Israeli society. The author describes the impediments that religious law poses to same-sex couples and their children, whose religious status is in question and who are not allowed to marry. Nevertheless, despite the excluding practices of Orthodox authorities, the Jewish ritual of circumcision for boys and traditional childbirth celebrations for girls are held and constitute moments in which relationships are reaffirmed. The author argues that the social networks attending these events and the performance of the circumcision by a religious officiant (mohel) convey the message that these families are an authentic part of the Jewish-Israeli collective, notwithstanding rabbinic opposition.

Lesbian mothers have received less research focus in Israel than gay fathers. Two comparative studies focused chiefly on the lack of differences between lesbian and heterosexual mothers. In the first, 30 women in two-mother lesbian families were compared with 30 two-parent heterosexual mothers on psychological distress, well-being, parental distress, and social support. No differences between the groups were found after controlling for socio-demographic differences [73]. In the second, Shenkman [74] matched 57 lesbian mothers with 57 heterosexual mothers and studied the association between basic need satisfaction in the couple relationship, namely the support the individual receives from the other person in the relationship for their sense of autonomy, competence, and relatedness, and its association with personal growth, as a function of sexual orientation. While results showed no differences between the two groups on these variables, a significant positive association between basic need satisfaction in the couple relationship and personal growth was found only among lesbian mothers. The author interprets this finding in terms of the specific features of lesbian couplehood, such as high emotional support, egalitarian division of labor, and the lack of traditional gender roles, which may contribute to a sense of personal growth in the context of lesbian motherhood, which is intentional, premeditated, and often realized after contending with minority stress.

Other studies have investigated the life experience of lesbian mothers in Israel. Ben-Ari and Livni [75], studied the constructed meanings that both biological mothers (who conceived through sperm donation) and nonbiological mothers attributed to their motherhood experience among eight lesbian couples who were parenting one to three children. Although the couples reported appreciating a sense of equality in their relationship, the birth of a child formed two different statuses of motherhood, a biological mother and a nonbiological mother, a distinction with legal and social consequences. In another study, interviews were conducted with 40 women in planned lesbian families in Tel Aviv [76] regarding their experience of motherhood. The findings suggest that these women struggle with an added stress to those included in the minority stress model: the burden to prove to themselves and to predominantly heterosexual society that they are accomplished and worthy mothers. The authors term this the “burden of proof,” reporting that it was manifested in a need to demonstrate excellence in mothering, the pressure to raise children who are both brilliant and “normative” (i.e., heterosexual and cisgender), and the sense of a duty to serve as role models for the LGBTQ community.

In another qualitative study that examined the disassembling of same-sex families, interviews were conducted with eight lesbian and gay parents from diverse family configurations (i.e., gay fathers through adoption, gay fathers through cross-border surrogacy, lesbian mothers through donor insemination, and co-parenting) [77]. The author contends that the disassembling of LGBT parent families offers a particular setting for exploring untraditional perceptions and enactments of kinship and family. Tracing separation processes and custody arrangements of same-sex couples with children indicated that at the family formation phase, couples side-lined genetic relatedness and emphasized social kinning. However, as the partners’ relations worsened, parent–child genetic links were gradually prioritized. Moreover, post-separation arrangements varied, with some couples continuing the former family contexts and others marginalizing non-genetic relatedness. The author concludes that having a genetic offspring seems to be an important determinant of post-separation relations.

Notably, little research has been conducted in Israel on children’s psychosocial adjustment as a function of parental sexual orientation. In the one study we found, Shechner, Slone, Lobel, and Shechter [78] compared the emotional and social development of children in four different family models: 15 families headed by single lesbian mothers; 21 two-mother lesbian families; 16 families headed by single heterosexual mothers by choice; and 24 two-parent heterosexual families. The analysis was based on mothers’ reports of children’s behavioral adjustment and children’s reports of peer relations and perceived self-competence. Results showed that children of lesbian mothers reported more prosocial behaviors and less feelings of loneliness than children from heterosexual parented families. No differences appeared in perceived self-competence across the family types. The authors conclude that the mother’s sexual orientation did not negatively affect the children’s adjustment. However, single parenthood, irrespective of sexual orientation, was associated with greater complications for children, displayed by more aggressiveness and externalizing behavior problems. Table 1 summarizes the main results and implications of the reviewed research on LGBTQ parent families in Israel.

## 5. Discussion: What Remains to Be Explored

Diverse social and cultural contexts create different realities for LGBTQ parent families, as well as influence the approaches and possibilities for research on this population. In the current review, we focused on investigations conducted in the Israeli sociocultural environment and related to its political climate, legislation, religion, and attitudes towards familism and natalism. In line with the cross-cultural theoretical perspective [16,79], our review suggests that the sociocultural context of Israel, including its pronatalist and familistic orientation, Orthodox Jewish law that disapproves homosexuality, and strong patriarchal norms, may play an important role in shaping the experiences of LGBTQ parent families. These experiences may include the difficulties faced by the parents in these families due to the discrimination they encounter and their struggles in the process of becoming parents, as well as their elevated levels of well-being when overcoming barriers to parenthood and feeling socially accepted in a society that sanctifies parenthood. As evidenced by the fact that most of the studies in the review were published in the past decade (92%), this field of research has seen dynamic growth in Israel in recent years, spurred by social transformations and legal advances that are creating a society that is more including and accepting of LGBTQ parent families. This avenue of research can therefore be expected to flourish in the future.

The review reveals that studies on LGBTQ parent families in Israel thus far have only examined the experiences of cisgender gay fathers and lesbian mothers, as no investigations of bisexual, transgender, or queer parents were found. There is therefore a need for future research that focuses on the experiences of bisexual, pansexual, transgender, and gender-nonconforming parents. Furthermore, the review demonstrates the importance of taking into account the sociocultural context in which studies are conducted. Thus, another valuable direction for future studies would be to examine how the experience of LGBTQ parent families in Israel compares with that of those in other countries and social contexts. Such cross-national studies would benefit from incorporating instruments assessing local cultural variables, such as attitudes toward natalism, voluntary childlessness, familism, and LGBTQ parenthood. Moreover, the review shows that relating to data derived from both qualitative and quantitative studies can paint a more comprehensive and authentic picture of the reality of LGBTQ parent families. Longitudinal studies, which explore the experience of LGBTQ parents and their children throughout the family life cycle, have yet to be conducted in Israel. These studies could add a developmental perspective regarding the characteristics, possible struggles, and coping strategies of the members in these families. In addition, existing studies have relied solely on self-reports. We thus recommend the use of multiple informants, particularly in studies focusing on children, in order to obtain a deeper understanding of the experience of these families. As noted above, studies on the children of gay and lesbian parent families in Israel are quite rare, indicating the need to expand this avenue of research. We fervently join other scholars in calling for more attention to be paid to family processes characterizing LGBTQ parent families, which may be associated with child adjustment [14,39]. Such processes may include the impact of parents’ socialization of assisted conception, self-efficacy, diverse family structures, and exposure to homophobia on the well-being of their children. In a similar vein, tapping children’s own views on their family model, as well as their perception of their origins, could increase our understanding of the experience of children in LGBTQ parent families and the factors that shape it.

Same-sex marriage is not yet possible in Israel. This legal vulnerability creates a hostile environment that may adversely affect sexual minorities [7]. Future research might relate to how gaining (or not gaining) marriage equality affects LGBTQ parent families, especially in regard to relationship commitment, divorce, and children’s relationships with their parents and other family members.

Most of the comparative designs reviewed in this paper consisted of a comparison between gay or lesbian parents and heterosexual counterparts. This between-difference approach should also be accompanied by a within-difference approach, which could shed light on the more nuanced similarities and differences between the diverse family configurations that come under the broad umbrella of LGBTQ parent families. These families may differ from one another on a number of sociodemographic characteristics as well, including race/ethnicity, social class, physical health, and geography (e.g., urban vs. rural residence). Therefore, the complex juxtaposition of multiple minority stressors (e.g., racism, heterosexism, cisgenderism, and disability status) also warrants further research attention.

Moreover, when investigating between-group differences, special attention should be paid to possible socio-demographic differences between LGBTQ and heterosexual parent families, such as in educational level and/or financial well-being, as these may be linked with parents’ health and mental health outcomes [80]. Comparative designs between lesbian/gay parents and heterosexual parents in Israel have found that gay parent families reported having fewer and younger children in comparison to heterosexual parent families, alongside higher economic status, e.g., [59,62,64]. Yet, whenever socio-demographic differences between the groups emerged, they were controlled in the statistical analyses to avoid confounding effects. Nevertheless, further research is needed to explore the contribution of socio-demographic variables (e.g., economic status, education, religiosity, residence place) to the mental health of the parents and children in LGBTQ parent families.

Additional attention should also be paid to differences between LGBTQ and heterosexual parents in parental motivations when conducting comparisons between these groups of parents. As mentioned earlier in the paper, for most LGBTQ individuals, parenthood is planned, deliberate, and intentional, whereas for heterosexual individuals, parenthood may be a default or a result of contraceptive failure. Therefore, future studies are recommended to compare LGBTQ parents with heterosexual parents with strong parenthood motivations, or with the need for assisted reproduction technologies. By doing so, they may reduce biases related to differences between the groups in parental motivation.

Future studies focusing on LGBTQ parent families should pay attention to possible methodological biases relating to individual social desirability and parental social desirability. LGBTQ parents may feel they need to prove to predominantly heterosexual society that they are accomplished and worthy parents, e.g., [76], which might be linked with socially desirable responses that may bias results. Therefore, we recommend that future studies comparing LGBTQ with heterosexual parent families should measure and control for individual and parental social desirability, alongside other possible biases such as in the selection of participants [81,82,83].

Finally, as more LGBTQ individuals become parents, they may seek therapeutic outlets designed to meet their unique needs. A special clinic was established in Israel in 2020 that offers guidance and counseling for LGBTQ parents, families, and prospective parents, as well as access to information on relevant legal issues. Research on such resources could shed light on the particular needs of this population from the health and mental health systems and could help in providing more sensitive and tailored services to LGBTQ parent families [84]. This, in turn, could promote the well-being of these families and enable them to thrive.

Notably, in this review, we adopted a more narrative approach to summarize the main findings, as this appeared to be the best way to present the results from disparate qualitative and quantitative studies, as well as those pertaining to the Israeli sociocultural and legal context. Thus, although we followed the main steps for a systematic review (e.g., premediated search strategy, inclusion, and exclusion criteria), this is not an orthodox systematic review, a fact which should be taken into consideration as it may compromise replication of the exact same search that has yielded the current findings [85]. Another limitation to consider is that we did not look at differences within LGBTQ parent families in Israel based on sociodemographic variables such as ethnicity, religiosity, and socioeconomic status. Future reviews of research might benefit from outlining the possible effects of these variables on the parenthood aspirations of LGBTQ individuals and the well-being of parents in LGBTQ families. In a similar vein, we have only searched for studies published in English or Hebrew, which could have resulted in a language bias by omitting studies on Israeli LGBTQ parent families that have been published in other languages. A further limitation of this review is that we did not include unpublished dissertations or master theses in our review. While this is a common exclusion criterion, we acknowledge the fact that valuable results may also exist in unpublished dissertations and master thesis.

To conclude, this review showed that while Israeli LGB individuals reported strong parenthood aspirations, they were less likely than their heterosexual counterparts to report a desire for parenthood. Possibly, this is due to the stigma related to LGBTQ parenthood. Moreover, in the pronatalist and familistic climate of Israeli society, success in becoming a parent, especially after contending with difficulties, seems the main road to social acceptance and a sense of triumph, which may explain many of the positive well-being outcomes that emerged among lesbian and gay parents. Our review also highlights the challenges lesbian and gay parents may face in Israel, such as the burden to prove to themselves and to society that they are accomplished and worthy parents, the need to deal with overseas pregnancies, and to cope with the duality between social acceptance and subversion under conservative gender and parental roles. We believe our review of existing studies, along with the directions we suggest for future research, can advance our knowledge of LGBTQ parent families. Given the rapid social change that Israel is currently undergoing, this sociocultural context is a particularly interesting arena for such research [86]. Based on the literature reviewed, and especially the findings showing a connection between lesbian and gay parenthood and positive psychological outcomes, we advocate for continuing to secure LGBTQ rights in Israel, both from a psychological and a human rights perspective. Policymakers and legislators should promote supportive and inclusive policies for LGBTQ parent families. While legislation regarding gay men’s access to surrogacy services is starting to change, adoption opportunities are still scarce, and reducing the stigma upon LGBTQ parenthood is much needed.

## Figures and Tables

**Table 1:** Summary table of the main results and implications of the review of research on LGBTQ parent families in Israel.

	**Main Results**
Parenthood aspirations among childless LGB individuals	A significant gap was found between strong fatherhood desires and low likelihood estimations among childless gay men [38]; quantitative design, *n* = 183. Lesbian women and gay men reported less desire to be parents than heterosexual counterparts [49]; quantitative design, *n* = 883. Lesbian women and gay men reported a lower estimated likelihood of parenthood than their heterosexual counterparts, which was also associated with adverse mental health outcomes [46]; quantitative design, *n* = 449. LGB individuals reported higher anticipation of stigma upon parenthood than their heterosexual counterparts, which also mediated the association between belonging to a sexual minority and lower parenthood aspirations [48]; quantitative design, *n* = 612. Lower parenthood aspirations mediated the association between being an LGB individual and adverse mental health outcomes [55]; quantitative design, *n* = 527. LGB participants from Israel and Portugal reported higher levels of parenthood desire, intent, and concern about childlessness than participants from the United Kingdom [56]; quantitative design, *n* = 504.
Israeli lesbian and gay parent families	Gay fathers reported higher levels of subjective well-being than heterosexual fathers [59]; quantitative design, *n* = 180. Gay fathers reported higher levels of subjective well-being and life meaning, as well as lower levels of depression, than gay men who were not fathers [60]; quantitative design, *n* = 90. Among gay fathers, but not among heterosexual fathers, higher self-perceived parental role was linked to higher life meaning [62], and less adverse mental health indicators of depression, negative emotions, and neuroticism [19]; quantitative designs, *n* = 164 in each study. Personal growth was higher among middle-aged and older gay fathers than among heterosexual fathers. Moreover, personal growth and purpose in life were higher among gay fathers than among gay men who were not fathers [63]; quantitative design, *n* = 300. Gay fathers through surrogacy reported greater satisfaction with parenthood, greater satisfaction with their lives, and higher levels of extraversion than heterosexual fathers [64]; quantitative design, *n* = 217. Main motivations for creating a hetero-gay family included the belief in biological connection to the child, and the belief that the child’s best interests dictate having two different-sex parents [20]; qualitative design, *n* = 9. Emotional experience of pregnancy among gay men through surrogacy abroad included frustration and concern due to their distance from the pregnant woman [67]; qualitative design, *n* = 16, alongside bureaucratic hardships involved in transnational surrogacy [68]; qualitative design, *n* = 20. Gay parenthood undermines existing concepts of parenthood, especially the essentialist notion of motherhood as a social construct [24]; qualitative design, *n* = 39. Gay fathers through surrogacy living in the periphery of the country challenge the dominant monolithic perception of this area as a locus of homophobia [70]; qualitative design, *n* = 14. The introduction of surrogacy as a medical option that can be legally pursued overseas, shaped a new couple-based fatherhood script [71]; qualitative design, *n* = 60. The performance of circumcision by a religious officiant (mohel) convey the message that lesbian and gay parent families are an authentic part of the Jewish-Israeli collective [72]; qualitative design, *n* = 65. No differences were found between lesbian and heterosexual mothers on psychological distress, well-being, parental distress, and social support [73]; quantitative design, *n* = 60. Positive association between basic need satisfaction in the couple relationship and personal growth was found only among lesbian mothers and not among heterosexual mothers [74]; quantitative design, *n* = 114. Among lesbian mothers, the birth of a child formed two different statuses of motherhood, a biological mother and a nonbiological mother, a distinction with legal and social consequences [75]; qualitative design, *n* = 16. Lesbian mothers reported on a specific stress related to a burden to prove to themselves and to heterosexual society that they are accomplished and worthy mothers [76]; qualitative design, *n* = 40. Among lesbian and gay couples with children, parent-child genetic links were gradually prioritized when partners’ relations worsened or post separation [77]; qualitative design, *n* = 8. Children of lesbian mothers reported more prosocial behaviors and less feelings of loneliness than children from heterosexual parented families [78]; quantitative design, *n* = 52.
	**Main Implications for Policy Makers and for Researchers**
	Policy makers should develop interventions aimed at reducing negative attitudes toward LGBTQ parenthood Policy makers should facilitate LGBTQ individuals’ access to the diverse paths to parenthood Researchers should take into consideration the sociocultural contexts when studying LGBTQ parent families and parenthood aspirations Researchers should also focus on the experiences of bisexual, pansexual, transgender, and gender nonconforming parents Researchers should promote more cross-national studies on LGBTQ parent families Researchers should conduct more longitudinal studies and reports by multiple informants, as such studies are lacking and are recommended Researchers are encouraged to conduct studies on the family processes and the adjustment of children raised in gay and lesbian parent families in Israel, as these are currently rare Researchers should further study the similarities and differences between the diverse family configurations that come under the broad umbrella of LGBTQ parent families Researchers should study the particular needs of LGBTQ parent families from the health and mental health systems

## Data Availability

The data presented in this paper are available on all the reviewed papers that were retrieved from the databases describes in the Methodology section.
